# Unmarried male migrants and sexual risk behavior: a cross-sectional study in Shanghai, China

**DOI:** 10.1186/1471-2458-13-1152

**Published:** 2013-12-09

**Authors:** Ke-Wei Wang, Jun-Qing Wu, Hong-Xin Zhao, Yu-Yan Li, Rui Zhao, Ying Zhou, Hong Lei Ji

**Affiliations:** 1 School of Public Health of Fudan University, 138 Dong An Road, Shanghai, P.R. China; 2 Department of Epidemiology and Social Science on Reproductive Health, Shanghai Institute of Planned Parenthood Research/WHO Collaborating Centre for Research in Human Reproduction Unit of Epidemiology, 2140 Xie Tu Road, Shanghai, P.R. China; 3 The Key Laboratory of Family Planning Device of National Population and Family Planning Commission, 2140 Xie Tu Road, Shanghai, P.R. China; 4 Ge Lan Su Shi Ke Company, Shanghai, P.R. China

## Abstract

**Background:**

In China, there is increasing concern because of the rapid increase in HIV infection recorded over recent years. Migrant workers are recognized as one of the groups most affected. In this study, HIV/AIDS-related knowledge, attitudes, and behavior among unmarried migrant workers in Shanghai are investigated, with the aim of providing critical information for policy makers and sex educators to reinforce sexual health services and sex health education targeting the behavior and sexual health of unmarried male migrants.

**Methods:**

A cross-sectional survey was conducted among unmarried male migrant workers in Shanghai, China’s largest city and housing the most migrants. A self-administered, anonymous questionnaire was used to collect information on knowledge, attitudes, and behavior associated with increased risk of HIV/AIDS.

**Results:**

A total of 2254 subjects were questioned, with a response rate of 91.3%. Among those interviewed, 63.5% reported sexual activities. Misconceptions regarding HIV transmission, poor perception of HIV infection, and low use of condoms were not uncommon. Among those who had sexual intercourse, 73.7% had not used condoms in their last sexual intercourse, and 28.6% reported having engaged in sexual risk behavior (defined as having at least one non-regular partner). Multivariate logistic regression analyses identified several indicators of sexual risk behavior, including younger age at first sexual intercourse (OR: 0.67, 95% CI: 0.31–0.91 for older age at first sexual intercourse), more cities of migration (OR: 2.91, 95% CI: 2.17–3.81 for high level; OR: 1.15, 95% CI: 1.06–1.29 for medium level), poor perception of acquiring HIV/AIDS (OR: 1.52, 95% CI: 1.33–1.96 for unlikely; OR: 2.38, 95% CI: 1.61–3.70 for impossible), frequent exposure to pornography (OR: 0.33, 95% CI: 0.11–0.43 for never; OR: 0.69, 95% CI: 0.60–1.81 for less frequently), not knowing someone who had or had died of HIV/AIDS and related diseases (OR: 2.13, 95% CI: 1.70–2.53 for no), and having peers who engaged in sex with a non-regular sex partner (OR: 4.40, 95% CI: 3.37–5.56 for yes).

**Conclusions:**

Today, it is necessary to reinforce sex health education among unmarried migrants and sexual health services should target vulnerable migrant young people.

## Background

It is clear that China is facing a frightening epidemic of AIDS. By 2011, the official estimate of the total number of people infected with HIV was 650,000 [1]. Although the epidemic remains has been largely confined to high-risk groups, the virus has begun to spread to an increasing number of areas and populations, resulting in the early phase of a likely the widespread dissemination of HIV to the general population [2]. At the same time, the mode of HIV transmission has shifted substantially over time. Sexual transmission has been the largest single cause of HIV infection in China, and heterosexual transmission has already become the main mode of transmission [1]. Rural-to-urban migration may be playing a crucial role in the rapid diffusion of HIV [3].

With China’s fast industrialization and urbanization since the initiation of the “open door policy” in the 1970s, China has experienced rapid economic growth. Large economic disparities between urban and rural areas led to the emergence of rural-to-urban migration as villagers looked for new jobs, higher income and homes in towns and cities. There were an estimated 230 million migrant laborers in China by the end of 2011 [4].

Nearly half of China’s migrants were born in the 1980s, their average age being about 28 years old [4]. The majority of migrants have a poor educational background. Although most are in the sexually active stage of their lives, they actually possess little knowledge about HIV, sex health-related matters, or protection, and have almost no access to preventive education or regular health care. This all leads to a major concern about the health consequences of migration in China today: migrant workers are particularly vulnerable to sexual risk behavior, which in turn makes them more susceptible to HIV infection [2,5,6].

Most young migrants are rural-to-urban migrants, far from home and released from the restraints exerted by their parents; they are living in single-sex dormitory housing and working long hours in difficult conditions; and their attitudes about sexuality and lifestyles have changed since they immigrated [2,5]. It is assumed that under such circumstances, these migrants are more likely to engage in sexual risk behavior, like having casual sex and paying for sex and having multiple sexual partners [7-9]. Some studies have shown that migrant workers’ knowledge of HIV/AIDS is minimal and that they are unable to obtain useful information, consultation, and healthcare services pertaining to HIV/AIDS [10,11]. Some researchers have found a relationship between sexual risk behavior and poor knowledge of HIV/AIDS. Sexual behavior studies in China and other countries have shown that poor knowledge of HIV/AIDS increases the likelihood of engaging in sexual risk behavior [12,13]. Studies from other countries have shown that the sexual attitudes of young, mobile people become increasingly open and that they are particularly likely to indulge in HIV/AIDS-related sexual risk behavior [14-17]. Similar studies of migrant workers in China have indicated that premarital and extramarital sex appear to have become more accepted among young people [18,19]. Hesketh [20] proposed that traditional attitudes to sexual relationships place migrant workers at low risk for engaging in casual sex. Na He [21] stated demand for legalization of commercial sex increases the risk of having more than one sexual partner. Xun Zhuang [22] concluded that acceptance of multiple sex partners increases the risk of engaging in sexual risk behavior. In addition, the household registration system (the *hukou*) has restricted peasant entry into the cities to control unwanted migration out of the countryside. Therefore, most rural-to-urban migrants can only gain temporary status in the cities and have to travel back to the countryside periodically. Several studies have shown that migrants tend to be at a higher risk of contracting HIV and other sexually transmitted diseases (STDs) than non-migrants [23,24]. A great many studies have indicated that HIV infected patients in China cities consist mostly of migrant workers [25-27]. Multiple sources have documented how migrants serve as a bridge population for the spread of HIV from migrant destinations back to native regions, with the frequent seasonal shifts between work in cities and return to homelands leading to the further transmission of HIV among their sex partners [28-30].

Previous investigations of migrants’ HIV-related sexual behavior in China have largely focused on male migrants or migrant female sex workers [21,22,31]. Few studies have focused on sexual risk behavior in rural-to-urban unmarried male migrants. Unmarried males in particular may be likely to engage in HIV risk-taking behavior, much more so than married males because of their specific physiological and psychological characteristics in the context of exposure to HIV risk-taking factors in the urban environment [2,32]. For this study, therefore, we conducted a community-based, cross-sectional study of HIV-related risk behavior among unmarried male migrants in Shanghai, China’s largest city with almost nine million migrants [33]. The objectives of the investigation were to describe the HIV/AIDS-related knowledge, attitudes, and risk behavior of and thus identify possible sexual risk behavior factors among unmarried male migrants in Shanghai.

## Methods

### Data

This study was conducted in Shanghai, the first largest and most highly migrant populated city of China. Located in the eastern part of the country, the city is administratively divided in to 16 administrative districts and one county. One (Xuhui district with 1.47 million people) of the districts, was randomly recruited, within which 12 communities were selected as sources of study participants.

A quota-sampling procedure was utilized to recruit a composite sample that was approximately proportionate to the overall distribution of the migrant population in 11 different occupational clusters (porter, operating a business stand, delivery man, decorator, installation worker, salesman, wholesale and retail trade, security guard, construction, manufacturing, and waiter) accounting for approximately 93% of the migrants. The scene investigation was administered during April and May 2012, with a final total of 2468 unmarried male participants volunteering to participate in the study after their informed consent had been obtained. Inclusion criteria for eligible participants were as follows: (1) Men; (2) Unmarried; (3) Not registered as a permanent resident of Shanghai; (4) Had resided in the current city for at least half a year.

The questionnaire for this study was designed mainly on the basis of the scales used in China’s National HIV Surveillance Surveys, and information was gathered from several group discussions with migrant workers. The measures used were in accord with others employed in other studies in China of knowledge/awareness about HIV/AIDS [21,22,34]. The feedback from group discussions suggested that the majority of migrant workers would respond more honestly about their sexual activities with an anonymous questionnaire. The questionnaire covered three main areas: The first part included less sensitive questions, such as the demographic characteristics of the migrant workers, peer influence, exposure to pornography, and acquaintance with someone who had or had died of HIV/AIDS and related diseases. The second part included their knowledge of and attitudes to HIV/AIDS. The third part consisted of questions regarding the respondents’ sexual activities and use of condoms.

### Measures

#### Socio-demographics

We included several socio-demographic characteristics: age (years), ethnicity, educational attainment, monthly income (dollars), number of cities of migration, age at first sexual intercourse, type of city housing, risk perception of acquiring HIV/AIDS, exposure to pornography, HIV/AIDS-related knowledge, whether non-regular sex should be acceptable, whether permissive regarding premarital sex, whether condom use could prevent HIV/AIDS, whether or not knew someone who had or had died of HIV/AIDS and related diseases, and whether or not have peers who had engaged in sex with a non-regular sex partner. In this study migrants’ peers only refer to friends and workmates around. Age was grouped into 4 categories: <15, 15–19, 20–24, and 25 years or older. Education was analyzed in terms of the following categories: elementary school or lower, junior high school, and high school or above. Ethnicity was grouped into Han and other. Monthly income (dollars) was grouped into 4 categories: <161.5, 161.6–322.9, 323.0–484.5, and 484.6 dollars or more. The number of cities of migration was stratified into 1, 2 and 3 or more. Age at first sexual intercourse was grouped into two bands: < 22 and 22 years or older. The housing type/place of residence was divided into 5 categories: collective dormitory with others, own room in a dormitory, room with parents, rented room, and other. Exposure to pornography was categorized into 3 groups: frequent (≥ 3 times every 6 months), less frequent (< 3 times every 6 months), and never. Peer influence was classified according to whether or not the migrant had peers who engaged in sex with a non-regular sex partner.

#### HIV/AIDS-related knowledge and attitudes

HIV/AIDS knowledge—the subscale score for HIV/AIDS knowledge (range 0–10)—was created by calculating the correct responses to ten items covering modes of transmission and protection information. Correct answers were credited with a score of one and incorrect answer with a score of zero, higher scores thus indicating a higher level of knowledge. The internal consistency of the subscale was 0.81. A summary score was developed from the ten HIV knowledge questions (three questions were about the three transmission modes of HIV, five on misconceptions about HIV transmission, one on whether regular use of antibiotics could prevent HIV/AIDS, and one on whether HIV/AIDS was curable). Participant’s attitude toward sexual behavior was evaluated by three items, namely, their agreement with the following statements: (1) “It is permissible for people to have premarital sex,” (2) “Casual sex should be acceptable,” and (3) “Do you think you are at risk of HIV infection?” (with the answer options “very possible,” “unlikely” and “impossible”). For condom knowledge and attitude, subjects were asked whether they regarded condom use as a good contraceptive measure and if they thought condom use could effectively prevent HIV/AIDS. Response options were either Yes or No.

#### Sexual behavior and condom use

Sexual risk behavior was defined as sex (heterosexuality by vagina) with a non-regular partner (casual or commercial sex partner) during the six months prior to the survey. Casual sex was defined as having sexual contact, without pay, with another person with no plans for a further long term/committed relationship with that person. Sexual behavior was assessed by questions regarding: (1) the relationships of sexual partners in the six months before the survey, and (2) condom use with sexual partners during the last sexual intercourse (yes/no). The outcome variables analyzed were four categorical variables related to the relationships with sexual partners: friends, living together, commercial sexual partners and casual partners. Casual and commercial sex partners were defined as non-regular, with friends and people they lived with coded as regular partner.

### Interviews

Eligible individuals were asked to complete a self-administered questionnaire in an isolated room or a quiet place to minimize embarrassment related to discussion of sensitive topics. Reading assistance was provided to participants with limited literacy. The study protocol was approved by the Ethics Committee of Fudan University School of Medicine. All study participants gave written consent before participation and all information was collected confidentially.

### Data analysis

Data on all the questionnaires were entered twice by different professionals using EpiData 3.1 to enable a comparison between the data and the correction of data entry mistakes. Data cleaning included consistency verification for all variables. The analysis was done using SAS version 9.2 (SAS Inc. Cary, NC, USA). Descriptive statistics included mean, frequencies, and proportions. HIV/AIDS-related knowledge was used as the continuous variable and entered with other independent variables with a statistical significance of < 0.10 in the univariate analyses into the logistic regression model. Non-regular sexual intercourse (yes/no) was used as the dependent variable. The variables were then removed from the equation one at a time using stepwise backward elimination until the removal did not lead to a significant decrease in the strength of the equation. Pearson correlation coefficients were < 0.30 for all bivariate relationships tested for the independent variables. No colinearity was found between the independent variables. Odds ratios (OR) were used as indictors of the strength of association. *P* < 0.05 was considered statistically significant.

## Results

### Characteristics of participants

Of the total of 2468 migrants approached to participate in the survey, 214 (8.7%) declined or were excluded because they did not provide information on sexual behavior, leaving a final sample of 2254. Of these, 65.3% reported sex experiences; over three-quarters (77.2%) were 15-24 years old, and the majority (96.1%) of Han ethnicity. Most (90.0%) had completed junior high school or above. Nearly half (46.6%) of the respondents reported that they had only migrated to one city. More four-fifths (83.9%) of the participants reported that their monthly income was less than 484.6 dollars, with the average monthly income at 303.9 dollars. Approximately two-fifths (38.5%) of respondents lived in a shared dormitory, 30.1% of the respondents had their own room in a dormitory, and 6.0% of the respondents lived in a rented room (Table 1).

**Table 1:** Demographic characteristics of study participants and HIV/AIDS-related knowledge and attitudes among study participants

**Variable**	**N = 2254**	**%**
Sex experience		
Yes	1472	65.3
No	782	34.7
Age (years)		
< 15	36	1.6
15–19	561	24.9
20–24	1143	50.7
≥ 25	514	22.8
Ethnicity		
Han	2165	96.1
Other	89	3.9
Educational attainment		
Elementary school or lower	230	10.0
Junior high school	1192	52.3
High school or above	832	37.7
Number of cities of migration		
1	1055	46.6
2	714	31.2
≥ 3	485	22.2
Monthly income (dollars)		
< 161. 5	394	17.1
161.6–322.9	803	35.7
323.0–484.5	695	31.1
≥ 484.6	362	16.1
Type of city housing		
Collective dormitory with others	878	38.5
Own room in a dormitory	670	30.1
Room with parents	478	21.3
Room rented	136	6.0
Other	92	4.1
HIV/AIDS-related knowledge (mean)	4.12	
Whether permissive regarding premarital sex		
Yes	1817	80.6
No	437	19.4
Whether non-regular sex should be acceptable		
Yes	904	40.1
No	1350	59.9
Risk perception of acquiring HIV/AIDS		
Very possible	399	17.7
Unlikely	735	32.6
Impossible	1120	49.7
Whether non-regular sex should be acceptable		
Yes	904	40.1
No	1350	59.9
Risk perception of acquiring HIV/AIDS		
Very possible	399	17.7
Unlikely	735	32.6
Impossible	1120	49.7

### Knowledge of and attitudes to HIV/AIDS

Most participants (73.5%-83.0%) could correctly identify the three major modes of HIV transmission, but many had substantial misconceptions regarding HIV transmission (data not shown). For example, nearly three-fifths (57.2%) thought that HIV can be contracted through mosquito bites, and one-third (34.2%) thought that it could be transmitted by eating with an HIV-positive person (data not shown).

Over four-fifths (80.6%) of participants agreed with the statement “It is permissible for people to have premarital sex,” and two-fifths (40.1%) agreed that “non-regular sex should be acceptable in China.” Half (50.3%) perceived themselves to be at risk of HIV infection, while 49.7% of respondents felt that they would not be likely to contract HIV/AIDS in the future, including those engaging in sex with non-regular partners. Regarding condom knowledge, approximately three-quarters (73.9%) of respondents regarded condom use as a good contraceptive measure, although only 43.0% knew that condom use could effectively prevent HIV/AIDS (Table 1).

### Sexual behavior among participants having sex

Table 2 shows the percentage distribution of the respondents according to variables related to premarital sex. About half (46.9%) of the respondents experienced sex before their 22th birthday, and the mean age at first sexual intercourse was 22.3 years. Almost two-thirds (63.7%) of the sample reported having had only one sexual partner. Over a quarter (28.6%) of participants reported having had non-regular (commercial and casual) sex in the last six months. Only 26.3% (*n* = 387) of the participants had used condoms in their last sexual intercourse, meaning that 73.7% (*n* = 1085) did not. Of the respondents who had engaged in sexual risk behavior, just 33.9% (*n* = 143) reported condom use in their last sexual intercourse, meaning that 66.1% (*n* = 279) did not (data not shown). About 6.7% (*n* = 98) reported having had oral sexual intercourse, and ten (0.68%) participants reported anal sexual intercourse with their partners.

**Table 2:** Percentage distribution of respondents according to variables related to sex behaviors among participants having sex experience

**Variables**	**N = 1472**	**%**
Age of first sexual experience		
< 22	691	46.9
≥ 22	781	53.1
Number of sexual partners in one’s lifetime		
1	937	63.7
≥ 2	535	36.3
The relationships of sexual partners		
Regular partner (friends and living together)	1050	71.4
Non-regular partner (commercial sexual partners and casual partners)	422	28.6
Condom use in the last sexual intercourse		
Yes	387	26.3
No	1085	73.7
Oral Sexual intercourse		
Yes	98	6.7
No	1374	93.3
Anal sexual intercourse		
Yes	10	0.68
No	1462	99.32

### Sexual risk behavior

Among all participants, 422 (18.6%) reported sexual intercourse with at least one non-regular partner in the six months preceding the survey (Table 3); and 28.6% of participants having sex experience reported having non-regular sex (Table 2). About 24.3% (*n* = 358) of participants having sex experience reported having had sexual intercourse with commercial sex workers, but only 10.9% (*n* = 160) reported engaging in casual sex (data not shown). Among participants having sex experience, the occurrence of sexual risk behavior increased significantly with age (p < 0.0001), with 37.4% of men in the oldest age group (≥ 25 year old) having had sex with a non-regular partner (Table 3). In univariate analyses, our primary outcome, sexual risk behavior, showed significant associations with age, educational levels, number of cities of migration, age at first sexual intercourse, type of city housing, risk perception of acquiring HIV/AIDS, exposure to pornography, whether non-regular sex should be acceptable, whether knew someone who had or had died of HIV/AIDS and related diseases, and whether had peers who had engaged in sex with a non-regular sex partner (Table 3).

**Table 3:** Univariate analyses of factors associated with ever having had sex with a non–regular partner among participants having sex experience

**Variable**	**N = 1472**	**Risk sex**		***P-value***	**OR**	**OR (95% CI)**
		**Yes**	**%**			
Age (years)						
< 15	2	0	0			
15–19	360	77	21.4	<0.0001	1	
20–24	746	209	27.9		1.42	0.91–1.93
≥ 25	364	136	37.4		2.19	1.58–2.70
Educational attainment						
Elementary school or lower	115	42	36.5	0.023	1	
Junior high school	778	202	25.8		0.61	0.31–0.89
High school or above	579	178	30.7		0.77	0.55–0.97
Monthly income (dollars)						
< 161. 5	107	32	29.9	0.99	1	
161.6–322.9	549	156	28.4		0.93	0.81–1.07
323.0–484.5	480	137	28.5		0.94	0.85–1.04
≥ 484.6	336	96	28.5		0.94	0.83–1.06
Number of cities of migration						
1	688	173	25.0	0.015	1	
2	448	140	31.3		1.36	1.03–1.71
≥ 3	336	109	32.4		1.44	1.27–1.62
Age at first sexual intercourse						
< 22	691	232	460	<0.0001	1	
≥ 22	781	190	591		0.64	0.51–0.81
Type of city housing						
Collective dormitory with others	573	140	24.4	<0.0001	1	
Own room in a dormitory	417	148	35.2		1.68	1.27–2.13
Room with parents	333	104	31.2		1.41	1.08–1.77
Room rented	99	22	22.2		0.88	0.78–0.97
Other	50	8	16		0.59	0.45–0.71
Risk perception of acquiring HIV/AIDS						
Very possible	428	66	15.4	<0.0001	1	
Unlikely	542	158	29.2		2.25	1.78–2.75
Impossible	502	198	39.3		3.54	2.91–4.17
Exposure to pornography						
Frequently	672	229	34.1	<0.0001	1	
Less frequently	716	175	24.3		0.62	0.38–0.88
Never	84	18	21.4		0.53	0.31–0.76
Whether permissive regarding premarital sex						
Yes	1177	329	27.9	0.21	1	
No	295	93	31.5		1.19	0.90–1.57
Whether non–regular sex should be acceptable						
Yes	560	179	31.9	0.028	1	
No	912	243	26.6		0.77	0.61–0.97
Whether condom use could prevent HIV/AIDS						
Yes	553	171	30.9	0.138	1	
No	919	251	27.3		0.84	0.67–1.06
Whether or not knew someone who had or had died of HIV/AIDS and related diseases						
Yes	397	69	17.4	<0.0001	1	
No	1075	353	32.8		2.32	1.74–3.10
Whether or not have peers who had engaged in sex with a non–regular sex partner						
No	748	196	26.2	0.034	1	
Yes	724	226	31.2		1.28	1.02–1.60

All factors significant at p < 0.10 in the univariate analysis were included in the multiple logistic regression analysis. After controlling for potential confounding variables, these results indicated that having had non-regular sex was associated with earlier age at first sexual intercourse, more cities of migration, poor perception of acquiring HIV/AIDS, frequent exposure to pornography, not knowing someone who had or had died of HIV/AIDS and related diseases, and having peers who had engaged in sex with a non-regular sex partner (Table 4). Respondents who were older (> 22) at first sex were less likely (37%) to have a non-regular sexual partner than respondents who were younger (< 22) at first sexual intercourse. For all participants, number of cities of migration was positively associated with sexual risk behavior. Males who had migrated to three or more cities were 2.91 times more likely to engage in risky sexual intercourse than those having migrated to only one city. We also found a significant inverse relationship between risk perception and sexual risk behavior. This result suggests that respondents who perceive themselves to be only at low (unlikely and impossible) risk were significantly more likely (OR = 1.52 and OR = 2.38, respectively) to take part in sexual risk behavior. Compared to those who frequently viewed pornography, respondents who were never or less frequently exposed to pornography were less likely to report sexual risk behavior (67% and 31%, respectively). Knowing someone who had or had died of HIV/AIDS and related diseases demonstrated a strong protective association with sexual risk behavior. Respondents who had never known people with these diseases were 2.13 times more likely to engage in sexual risk behavior than were those who had known such people. Finally, respondents who had peers engaging in sex with a non-regular sex partner were 4.4 times more likely to be involved in sexual risk behavior than were those who did not engage thus.

**Table 4:** Logistic regression analysis of factors correlated with ever having had sex with non-regular sex partner

**Variable**	**OR**	**OR (95% CI)**	***P-value***
Age at first sex			
< 22	1		0.035
≥ 22	0.67	(0.31, 0.91)	
Number of cities of migration			
1	1		0.028
2	1.15	(1.06,1,29)	
≥ 3	2.91	(2.17,3.81)	
Risk perception of acquiring HIV/AIDS			
Very possible	1		<0.0001
Unlikely	1.52	(1.33,1.96)	
Impossible	2.38	(1.61,3.70)	
Exposure to pornography			
Frequently	1		0.007
Less frequently	0.69	(0.60, 0.81)	
Never	0.33	(0.11, 0.43)	
Whether or not knew someone who had or had died of HIV/AIDS and related diseases			
Yes	1		0.012
No	2.13	(1.70–2.53)	
Whether or not have peers who had engaged in sex with a non-regular sex partner			
No	1		<0.0001
Yes	4.40	(3.37–5.56)	

## Discussion

To our knowledge, this study is the first in China to document sexual risk behavior and attempt to assess the likely causal factors for sexual risk behavior among unmarried male migrants.

We found a high proportion of male migrants having sexual intercourse before marriage, which may still represent an underreporting due to the sensitive nature of the issue and strong social and cultural taboos related to this area in China [35]. At the same time, the prevalence of sexual risk behavior (defined as sex with a non-regular sex partner) among single men revealed by this data is much higher than the levels observed by Xun Zhuang et al [22]. This may be because the latter study was based on the general population rather than focusing on an unmarried group. In addition, among young unmarried men interviewed in this survey, the rate of sexual risk behavior increased significantly with age. This means older respondents were more likely than younger ones to have non-regular sexual relationships. One of the main reasons could be that sexually active unmarried men in general engaged in sexual risk behavior in order to meet physical needs and relieve their loneliness and anxieties about home [2].

Of the participants engaging in premarital sex in our sample, 36.3% reported having had more than one sexual partner in their life, a proportion significantly higher than that reported by another study [21]. This finding is important because it enhances our understanding of the possible effects of multiple sex partners as increasing the likelihood of rapid transmission of HIV/AIDS. If someone has acquired HIV/AIDS, he may infect other partners, so not only are a large proportion of unmarried men at high risk of infections (those with multiple sex partners), but their partners are also likely to be at risk [36].

The study also showed that only about 43.0% of all migrants believed that use of condoms could prevent HIV/AIDS, and infrequent condom use, another high-sexual risk behavior, was common among the males who had had sex in the six months before the study period. Nearly three-quarters (73.7%) of respondents did not use a condom. More importantly, condom use was also quite low among the migrants who engaged in sexual risk behavior. A considerable proportion (66.1%) of the male migrants also did not use a condom in their last sexual intercourse. These results show that sexually active individuals do not routinely practice safe sex, leading to a high risk of HIV infection. The low rate of condom use may explain their poor knowledge about the protective effects of condoms against HIV [37]. The findings indicate that the government needs to pay more attention to interventions targeting unmarried male migrants that could be effective in improving their awareness of condom use. For example, community interventions, including lec-tubes (experts lecture), posters, brochures, and videos, could be used to help them think of condoms as a cost-effective approach to sexually transmitted diseases/HIV prevention and contraception.

Despite a majority of participants having heard of AIDS and knowing the major routes of HIV transmission, many had substantial misconceptions regarding other routes. This report is similar to reports by authorities citing knowledge of mode of transmission of HIV/AIDS among Nepalese migrants in India [9]. Here, about half of respondents did not perceive themselves to be at risk of HIV infection: also, respondents largely considered premarital sex to be acceptable. In our study, this phenomenon of risk behavior and perception of HIV/AIDS, which indicates a risk of HIV infection, highlights the need for the promotion of safer sex among migrants.

These study findings have important implications for developing HIV intervention programs targeting migrants in Shanghai. First, we found that, consistent with an earlier study [22], males that have first sexual intercourse at a younger age were more likely to report having non-regular sex partners in the previous six months, suggesting the importance of focusing on HIV prevention interventions on younger, sexually experienced males. Second, a noteworthy finding in this study was the strong association of sexual risk behavior with exposure to pornography. Those who were exposed to pornography were more likely to engage in risk behavior than those who were not. This suggests that more intense efforts should be made to improve awareness about the seriousness of exposure to pornography among unmarried males and help them to develop a proper attitude to it. Third, another noteworthy finding was that having peers who had engaged in sex with a non-regular sex partner increased the likelihood of the involvement of unmarried males in risky sexual encounters. Studies conducted in the U.S. have demonstrated that social network members can influence the behaviors of others through a variety of mechanisms, including social comparisons, social control processes, and fear of social sanctions [38,39]. In common with other types of human behavior, our results demonstrate that peers may exert a significant influence on the sexual risk behavior of individuals. The results are also in line with those of another study, which found that the social influence of peers contributes to increased sexual risk behavior among migrants [40]. The findings imply that HIV intervention programs not only focus on individual risk takers but also on their broader social network of peers. Fourth, our analysis revealed that the number of cities to which men migrated was positively associated with higher risk sex and that participants who had a history of moving to more cities of migration were more likely to report higher risk sex. Unique HIV prevention and intervention strategies should be conducted in this population. Fifth, as expected, participants who perceived themselves to be at low risk of HIV infection were more likely to report non-regular sexual partners than those having a higher risk perception. Despite many achievements in HIV knowledge dissemination, there remains an essential need to educate migrants more extensively about HIV/AIDS prevention and transmission, and to increase personal skills in protection against HIV infection. Sixth, we found the strong protective association with sexual risk behavior of knowing someone who had or had died of HIV/AIDS and related diseases. This finding was similar to the results of a study conducted among the young in the United States [41]. Interventions need to address ways to help migrants better understand the serious consequences of sexual risk behavior, including various infectious diseases and especially HIV/AIDS. Seventh, our study also showed that many migrants had substantial misconceptions regarding HIV transmission. Therefore, interventions to improve HIV/AIDS-related knowledge need to focus on unmarried male migrants, particularly those engaging in risky behaviors and emphasize the ways HIV is not transmitted in addition to the major routes of HIV transmission. Finally, more intervention programs should target migrants, especially young unmarried males, and condom use should be emphasized strongly, targeting also non-regular sex partners.

## Conclusions

To our knowledge, this study is the first in China to focus on unmarried male migrants, an understudied subgroup with a potentially greater risk of contracting HIV in China. Our study not only described HIV/AIDS-related knowledge, attitudes, and sexual risk behavior in this subgroup but also identified possible sexual risk behavior factors, such as earlier age at first sexual intercourse, more cities of migration, poor perception of acquiring HIV/AIDS, frequent exposure to pornography, not knowing someone who had or had died of HIV/AIDS and related diseases, and having peers who had engaged in sex with a non-regular sex partner. The results provide critical information for policy makers and sex educators to reinforce sexual health services and sex health education targeting the behavior and sexual health of unmarried male migrants.

### Limitations

This study has also several limitations. First, this was a cross-sectional survey, limiting our ability to draw causal inferences. Second, all variables were self-reported. Respondents may have underreported sexual risk behavior because sex is a sensitive topic in China. Third, due to the cross-sectional nature of the data, most responses were measured retrospectively, and recall bias might have occurred. Last, our sampling was limited to only a single city. We cannot, therefore, claim that our findings are representative for all unmarried male migrants in China—but the results do provide useful insights and suggest possible interventions to address sexual health risks for migrants.

## Competing interests

There is no conflict of interest and the authors alone are responsible for the content and writing of the paper.

## Authors’ contributions

KWW performed the statistical analysis, involved in interpretation of the results and drafted the manuscript. WQW, HXZ, YYL and RZ conceived of the study and participated in the design of the study. YZ and HLJ participated in its design and coordination and helped to draft the manuscript. All authors read and approved the final manuscript.

## Pre-publication history

The pre-publication history for this paper can be accessed here:

http://www.biomedcentral.com/1471-2458/13/1152/prepub
